# Treatment and Outcomes in Myocardial Infarction and Cancer—A German Real-Life Analysis

**DOI:** 10.1016/j.jaccao.2026.04.007

**Published:** 2026-06-16

**Authors:** Stefanos T. Papageorgiou, Jannik Feld, Christiane Engelbertz, Lena Makowski, Georg Karanatsios, Alicia J. Friedrich, Jeanette Köppe, Joachim Gerß, Christoph Schliemann, Holger Reinecke, Stefan A. Lange

**Affiliations:** a University Hospital Muenster, Cardiol, Department of Cardiology I - Coronary and Peripheral Vascular Disease, Heart Failure, Muenster, Germany; b University of Muenster, Institute of Biostatistics and Clinical Research, Muenster, Germany; c University Hospital Muenster, Cardiol, Department of Cardiology III - Adults with congenital heart disease, Muenster, Germany; d University Hospital, Muenster, Hematology and Oncology Department – Internal Medicine Clinic A, Muenster, Germany

**Keywords:** acute coronary syndrome, bleeding, coronary artery, electronic health records, epidemiology, outcomes, prediction, risk factor, treatment

**Figure:**
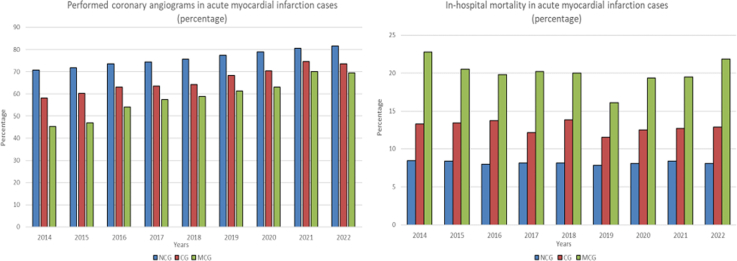

In recent years, the prevalence of cancer as a comorbidity among patients with myocardial infarction (MI) has increased and is associated with higher all-cause mortality and bleeding complications.^1^ Both diseases share important risk factors, including tobacco smoking, genetic factors, and obesity.^2^ In addition, cancer pathophysiology (proinflammatory and procoagulant states), specific treatment regimens (cardiotoxic immunotherapy and chemotherapy), and shared genetic factors (tumor suppressor genes like BRCA1/2) may further increase the risk of cardiovascular events. Conversely, cancer diagnoses after MI may also be more frequent because of shared risk factors and the detection of presymptomatic cancers during intensive diagnostic evaluation and follow-up after MI.^3^ To better assess trends in the care of patients with MI with and without cancer, we performed a retrospective cohort study of MI cases in Germany.

We included all MI cases between January 1, 2014, and December 31, 2022, identified by International Statistical Classification of Diseases and Related Health Problems (ICD) codes I21∗ or I22∗, together with demographic data, associated comorbidities, and interventions performed. Cancer status, including metastatic disease, was determined using the corresponding ICD codes C00∗ to C97∗. The primary endpoint was in-hospital mortality. Cases were stratified into three groups according to cancer status: NCG (no cancer group), CG (cancer group without metastases), and MCG (metastatic cancer group). The data were obtained from the German Federal Statistical Office (Statistisches Bundesamt [DESTATIS]; https://www.destatis.de).^4^ These data are based on the German reimbursement system, which requires coding of primary and secondary diagnoses for all hospital admissions. Diagnoses were coded using the International Statistical Classification of Diseases and Related Health Problems, 10th Revision, German Modification (ICD-10-GM), and diagnostic, endovascular, and surgical procedures were coded using the German procedure classification (OPS). The data were case based rather than patient based and were accessed remotely in an anonymized format.

The identified cancer types included breast, colorectal, lung, prostate, and urinary cancers; all remaining cancer types were grouped as “other cancers.” Hematologic malignancies were not included because of their heterogeneity in symptoms, treatment, and prognosis and because the conventional metastasis model does not readily apply to these malignancies. The analysis included all inpatient acute MI cases in Germany and did not represent a subsample. Crude annual MI case counts were calculated using the total number of MI cases in each group and the total population of Germany in each study year.^5^ The results in Table 1 are presented as absolute numbers, with percentages shown in parentheses. Analyses were performed using SAS version 9.4 (SAS Institute, Inc.) and R version 4.3.2 (R Core Team, 2024).^6^

**Table 1:** Crude Annual MI Case Counts and Selected Baseline Characteristics, Interventions, and In-Hospital Outcomes by Cancer Status, 2014-2022

	Parameter	NCG (N = 1,851,588)	CG (N = 26,508)	MCG (N = 7,583)
Baseline characteristics	Age, y, median (Q1–Q3)	71 (60-80)	76 (69-82)	75 (67-81)
	Male sex	1,239,379 (66.9)	18,471 (69.3)	5,197 (68.5)
	Diabetes mellitus	538,429 (29.1)	8,349 (31.5)	2,300 (30.3)
	CKD	392,629 (21.2)	8,496 (32.1)	2,170 (28.6)
	HF	711,678 (38.4)	12,871 (48.6)	3,664 (48.3)
Interventions	CAG	1,405,195 (75.9)	17,426 (65.7)	4,378 (57.7)
	DES/BMS	1,036,172 (57.5)	12,090 (45.6)	2,965 (39.1)
	CABG	99,857 (5.4)	1,182 (4.5)	151 (2.0)
In-hospital outcomes	In-hospital mortality	151,375 (8.2)	3,424 (12.9)	1,516 (20.0)
	blood/blood transfusion	219,917 (11.9)	6,726 (25.4)	1,861 (24.5)
	AKI	142,987 (7.7)	3,570 (13.5)	1,112 (14.7)


MI Cases Per Year	2014	2015	2016	2017	2018	2019	2020	2021	2022
NCG	215,300	215,102	215,177	213,724	208,487	208,877	194,623	192,578	187,720
CG	3,326	3,184	3,132	3,114	2,952	2,896	2,777	2,693	2,434
MCG	914	950	909	856	875	851	802	794	632

Values are n (%) unless otherwise indicated. Annual values are crude MI case counts.

AKI = acute kidney injury; CABG = coronary artery bypass grafting; CAG = coronary angiography; CG = cancer group without metastases; CKD = chronic kidney disease; DES/BMS = drug-eluting stent/bare-metal stent; HF = heart failure; MCG = metastatic cancer group; MI = myocardial infarction; NCG = no cancer group. Temporal Trends in Coronary Angiography and In-Hospital Mortality by Cancer Status.

Over the 9-year period (2014-2022), 1,885,679 MI cases were identified. Of these, 26,508 (1.4%) were in the CG, and 7,583 (0.4%) were in the MCG. Male sex accounted for approximately two-thirds of cases in each group. The median age was higher in the CG and MCG than in the NCG.

During the study period, annual MI case counts decreased in all three groups, with the greatest decline in the MCG (30.9%), followed by the CG (26.8%) and the NCG (12.8%). Coronary angiography increased across all groups over the study years, with the largest increase in the MCG (53.4%), followed by the CG (26.5%). In-hospital mortality across NCG, CG, and MCG was 8.2%, 12.9%, and 20.0%, respectively.

These findings suggest a paradox: Despite increased coronary angiography over time, in-hospital mortality did not decline correspondingly. Further research is needed to better understand this finding, ideally including postdischarge outcomes. The increase in coronary angiography may reflect the development and expansion of cardio-oncology and greater recognition of the elevated cardiovascular risk in these patients. The observed reduction in MI cases may be related to improved primary prevention in Germany, with earlier diagnosis of coronary disease in the outpatient setting and modification of risk factors.

Limitations of the study include the case-based, rather than patient-based, nature of the data, which introduces a risk of double counting and overestimation, as well as the retrospective observational design, which is subject to unmeasured confounding, including potential confounders such as medications and cancer therapies with differing cardiotoxicity risk. In addition, cancer activity and disease status (eg, refractory disease, partial or complete remission, or unclear response to therapy) could not be determined from the available data. Postdischarge mortality data were also unavailable.

Given the high incidence of cancer, particularly among older patients with comorbidities, and the observed disparities in outcomes, there is a need for further investigation in this area and for the development of tailored strategies for this patient population.

### Data Availability

All data used in this work are publicly available from the German Federal Statistical Office (DESTATIS, https://www.destatis.de). RDC of the Federal Statistical Office and Statistical Offices of the Federal States, DOI: [10.21242/23141.2014.00.00.6.1.0 to 10.21242/23141.2022.00.00.6.1.0], own calculations.

## Funding Support and Author Disclosures

The authors acknowledge support from the open access publication fund of the University of Münster. Dr Köppe reports research funding from the German Society for Trauma Surgery sponsored by Stryker, outside the submitted work. The authors have reported that they have no relationships relevant to the contents of this paper to disclose.
